# A Hybrid Lung and Colon Histopathological Image Classification Framework Using MobileNetV3-Small Deep Features and Differential Evolution Optimization

**DOI:** 10.3390/diagnostics16091256

**Published:** 2026-04-22

**Authors:** Muhammad Usama Naveed, Sohail Jabbar, Muhammad Munwar Iqbal, Awais Ahmad, Ibrahim S. Alkhazi, Mansoor Alghamdi

**Affiliations:** 1 Department of Computer Science, University of Engineering and Technology, Taxila 47080, Pakistan; 23-ms-ds-1@students.uettaxila.edu.pk (M.U.N.); munwar.iq@uettaxila.edu.pk (M.M.I.); 2 College of Computer and Information Sciences, Imam Mohammad Ibn Saud Islamic University (IMSIU), Riyadh 11432, Saudi Arabia; abmohmmed@imamu.edu.sa; 3 Department of Information Technology, College of Computer, Qassim University, Buraydah 52581, Saudi Arabia; i.alkhazi@qu.edu.sa; 4 Department of Computer Science, University of Tabuk, Tabuk 71491, Saudi Arabia; malghamdi@ut.edu.sa

**Keywords:** lung and colon cancer, histopathological imaging, deep learning, transfer learning, feature optimization, classification

## Abstract

**Background/Objectives:** Cancer remains one of the leading causes of mortality worldwide, with lung and colon cancers among the most prevalent. Conventional histopathological diagnosis is time-consuming, requires expert pathologists, and is susceptible to human error. **Methods:** To address these limitations, this study proposes an automated classification framework for lung and colon cancer using histopathological images. The proposed method employs a lightweight pretrained deep learning model, MobileNetV3-Small, through transfer learning. Training is performed on an enhanced version of the LC25000 dataset, in which redundant image patches are removed to improve robustness and clinical generalizability. The images were initially available in multiple resolutions, which are resized to 224 × 224 × 3 to match the canonical input size of MobileNetV3-Small. Deep features are extracted from the dropout layer as it provides regularized representation of high-level features by reducing the overfitting (dimension N × 1024), which are optimized using a differential evolution algorithm, reducing the feature space to N × 60. These optimized features are evaluated using multiple classifiers. **Results**: Experimental results demonstrate a maximum classification accuracy of 98.14% using a Quadratic Support Vector Machine (SVM) and a 21.3× speed-up achieved with bagged trees, outperforming several state-of-the-art approaches representing a 3.34% improvement over the baseline study on the enhanced dataset. **Conclusions:** The results confirm that the proposed framework effectively balances high accuracy with computational efficiency. The use of a lightweight deep model combined with feature optimization makes the approach well-suited for practical clinical environments.

## 1. Introduction

Globally, cancer continues to be a significant cause of death, with lung and colorectal cancers being prevalent. Lung cancer is the most common cause of cancer-related deaths worldwide and the second most common type of cancer overall [1]. Lung cancer accounted for 18% of all cancer deaths in 2020, with 1.8 million new cases caused by the disease [2]. Compared to other central malignancies, lung cancer had a significantly poorer 5-year survival rate (7–25%) [3]. Due to its high death rate, lung cancer’s mortality distribution closely matched its incidence distribution, resulting in a significant worldwide disease burden. It is notable that about 17 patients (0.54%) out of 3102 patients with lung cancer also received a concurrent diagnosis of colon cancer within one month, suggesting that these two cancers significantly overlap [4].

The respiratory system is severely hampered by lung cancer, which frequently manifests as symptoms including hemoptysis, shortness of breath, chest pain, and a chronic cough [5]. On the other hand, colon cancer mainly affects the digestive tract and frequently results in abdominal pain and rectal hemorrhage [6]. Even though non-invasive approaches such as radiography and Computer Tomography (CT) [7] are commonly used to detect lung and colon cancers, they are not always sufficient for a conclusive diagnosis [8]. Histopathological analysis of tissue samples remains the standard for precise diagnosis and therapy planning [9]. However, manual analysis by pathologists is labor-intensive, time-consuming, and prone to human error, especially in early-stage malignancies, where diagnostic signs may be subtle [10].

Technological developments in deep learning (DL) and artificial intelligence (AI) have created exciting prospects to automate cancer diagnosis, reduce pathologists’ burden, and enhance diagnostic accuracy [11]. convolutional neural networks (CNNs), in particular, have demonstrated an impressive ability to extract complex patterns from histopathology images [12]. Early cancer detection and multi-stage classification are enabled by these models’ ability to identify discriminative characteristics that human specialists might find challenging [13]. In addition to histopathology, DL has demonstrated significant enhancement in radiography, Magnetic Resonance Imaging (MRI), and retinal imaging, providing enhanced analytical precision and expedited decision-making [14]. Colorectal cancer is a significant global health concern, requiring accurate and efficient diagnostic methods. Histopathological image analysis has emerged as a reliable approach for early detection. In this work, we focus on improving computational efficiency and classification performance using deep learning and optimization techniques.

### 1.1. Related Work

For lung and colon cancer histopathological imaging classes, Ochoa-Ornelas, R. et al. [15] proposed a machine learning- and deep learning-based hybrid approach by using the two deep models along with gray wolf optimization. The original LC25000 was modified to increase robustness and clinical generalizability, as the original datasets lack generalizability due to image augmentation. To do so, a contrast-limited adaptive histogram equalization (CLAHE) technique was applied during preprocessing. Moreover, 1000 new images were added to three malignant classes, namely colon adenocarcinoma, lung squamous cell carcinoma, and lung adenocarcinoma, which were gathered from the NCI (National Cancer Institute) GDC data portal. The features were extracted and fused by transfer learning the MobileNetV2 and EfficientNetB3 models. Optimal feature selection was performed using GWO, and the data were split into 80:20 for classification. The proposed methodology achieved 94.8% accuracy using the MEGWO-LCCHC technique.

A DL-based classification framework for lung and colon classification using DL and digital image processing (DIP) was proposed by Masud, M. et al. [8]. They have analyzed and classified histopathological images of lung and colon tissues into three malignant and two benign classes. The image-sharpening-based technique, i.e., the unsharp masking, was used in the preprocessing phase. The features are extracted using two basic digital image processing techniques, namely the 2D Fourier and 2D wavelet transforms. The features from 2D fast Fourier transform (2D-FFT) and 2D Discrete Wavelet Transform (2D-DWT) are then fused to get the resultant feature vector. The results show that the proposed technique performs well, with an accuracy of 96.33%.

Mehmood, S. et al. [16] presented a technique for lung and colon cancer detection using class-selective image processing and transfer learning. LC25000, which comprises five classes for lung and colon cancer, was used in this study. Transfer learning was performed on a pretrained deep CNN, AlexNet, by tuning its four layers using the respective dataset. The results after transfer learning do not show strong performance, with the highest accuracy of 89%. A single class caused this degradation, so instead of applying preprocessing to the whole dataset, the author applies the contrast enhancement technique to the respective class only. This technique improves the overall classification accuracy by 9.4%, bringing it to 98.4%.

A lung cancer histopathological image classification framework assisted by the Non-Local Mean (NLM) was proposed by Kumar, A. et al. in [17]. In the preprocessing phase, the images are enhanced by eliminating the noise and preserving edges using the NLM filter. These denoised images are then passed to ML3CNet (a multi-headed convolutional neural network for lung cancer classification), which was proposed in this paper. To reduce model size, a quantization technique was used, enabling the model to run faster and use less memory. The classification results show that ML3CNet performed well, achieving an accuracy of 98.92%. Recent advances in the medical field, which include 3D-printing and IoT-enabled systems, have transformed cancer diagnosis and treatment. 3D printing allows histopathological and imaging data to be converted into physical anatomical models, supporting precise surgical planning, patient-specific treatment, and medical education. On the other hand, IoT technologies enable real-time data acquisition, remote monitoring, and cloud-based processing of medical images, facilitating efficient diagnostics.

The ${DS}^{2}{LC}^{3}Net$ was proposed by Ijaz, M. et al. [18], in which lung and colon classification were performed using transfer learning and optimization. Two pretrained deep CNN models, i.e., ResNet50 and EfficientNetB0, were fine-tuned, and their deep features were extracted. The extracted deep feature vector is then fused using a priority-based serial approach. The fused feature vector was then fed into the gray wolf optimizer (GWO) for optimal feature selection. For the final prediction, the author used a soft voting technique and achieved an overall accuracy of 98.73% on the ‘LC25000’ dataset, improving ‘prediction time’ by 19.14%. Naveed, M.U. et al. [19] proposed a lung cancer classification technique by transferring learning from the EfficientNetB3 model over the LC25000 dataset, excluding the colon classes. The deep features are extracted by utilizing the TL model and fed to the classifiers for comparative analysis. The result shows that the proposed methodology achieves an accuracy of 99.80%.

Anjum performed a lung cancer classification on a histopathological imaging dataset, as in S. et al. [20]. In this study, the author used pretrained variants B0-B7 of EfficientNet and transferred them to the LC25000 dataset at different image resolutions for each variant. The evaluation and analysis show that at a 260 × 260 pixel resolution, EfficientNetB2 achieved the highest accuracy of 97%. Kumar, N. et al. [21] presented a lung and colon classification technique by using handcrafted and dense feature extraction. The handcrafted features were extracted based on texture, shape, color, and structure, and transfer learning was performed across seven deep CNN architectures for feature extraction. This study achieved an accuracy of 98.60% over extracted deep features of DenseNet-121.

Oubaalla et al. [22] aim to increase accuracy by demonstrating the impact of combining transfer learning (TL) and ensemble learning techniques for lung and colon classification. In this article, three different deep models were transfer-learned, i.e., DenseNet, VGG, and ResNet, for feature extraction, based on the ensemble learning technique. The authors’ proposed method shows accuracies of 97%, 97.4%, and 97.5% on the training, testing, and validation data, respectively. Anusha, M. et al. [23] proposed a transfer learning-based technique in which the VGG16 model was trained on the LC25000 dataset. In this study, a rigorous preprocessing was performed to enhance the image quality. The proposed technique achieved 98.69% accuracy and an ROC-AUC score of 99.96%.

A lightweight deep convolutional neural network (CNN) was proposed by Sakr, A.S. et al. [24] for efficient colon cancer classification. The publicly available histopathological image dataset of colon cancer was normalized and fed to a deep CNN model, which depicted an accuracy of 99.50%. Togaçar, M.J.C.i.B. [25] trained a DarkNet-19 model from scratch and extracted the deep features by using this model. The inefficient features were selected using Manta Ray Foraging and equilibrium optimizers, which were then distinguished from the original feature set to obtain the efficient features. An accuracy of 99.69% was achieved by passing this feature set to SVM.

There is a lot of research that has already been done in the field of lung and colon cancer classification and detection [8,15,16,17,18,20,26,27]. These studies used various techniques, including preprocessing to enhance dataset images, transfer learning/fine-tuning, feature extraction, optimization, and deep learning to improve accuracy. As stated earlier, DL has shown tremendous potential in radiography, retinal imaging, and Magnetic Resonance Imaging (MRI), offering enhanced diagnostic accuracy [28]. However, a problem with deep learning is that it requires a large volume of data to achieve good accuracy [29]. In medical imaging, it can be difficult to collect such a large volume of data [30]. The evolution of transfer learning has had a tremendous impact on the field of computer vision, yielding strong results even on limited datasets [31]. Moreover, task-specific adaptation is achieved in transfer learning by modifying the layers of the pertained deep convolutional neural network (DCNN) models, such as ResNet, EfficientNet, DenseNet, VGG, and AlexNet, etc., to achieve good results, by most of the researchers. Although existing studies have achieved high classification accuracy, many rely on computationally intensive architectures and lack efficient feature optimization mechanisms. These limitations highlight the need for lightweight frameworks that maintain performance while reducing computational overhead. The proposed approach addresses these challenges by integrating transfer learning with an optimization-driven feature selection strategy.

### 1.2. Contribution

Early detection of lung and colon cancer is crucial, as they are some of the most common diseases [32]. To do so, we have proposed an automated classification of lung and colon cancer using optimal deep features. The dataset used in this study consists of 25,000 histopathological images gathered from [15], an enhanced version of the original LC25000 dataset proposed by Ochoa-Ornelas, R. et al., to improve robustness and clinical generalizability. This article primarily focuses on reducing prediction time while retaining or minimizing accuracy. The key contribution is as follows:

- Utilization of an enhanced histopathological dataset instead of the original LC25000 dataset to provide a more diverse and challenging evaluation setting.
- A transfer learning-based deep feature extraction framework is developed using the pretrained MobileNetV3-Small architecture, enabling efficient representation learning from histopathological images.
- An optimal feature selection strategy based on the differential evolution (DE) optimization algorithm is applied using a custom fitness function to the extracted deep features, reducing feature dimensionality while preserving discriminative information.
- A comparative classification framework is employed using multiple classifier families, including support vector machines (SVMs), neural networks (NNs), and decision trees (DTs), to evaluate the robustness of the selected features.
- Comprehensive performance evaluation across different stages of the pipeline to assess the effectiveness of each component.

## 2. Materials and Methods

**Figure 1:**
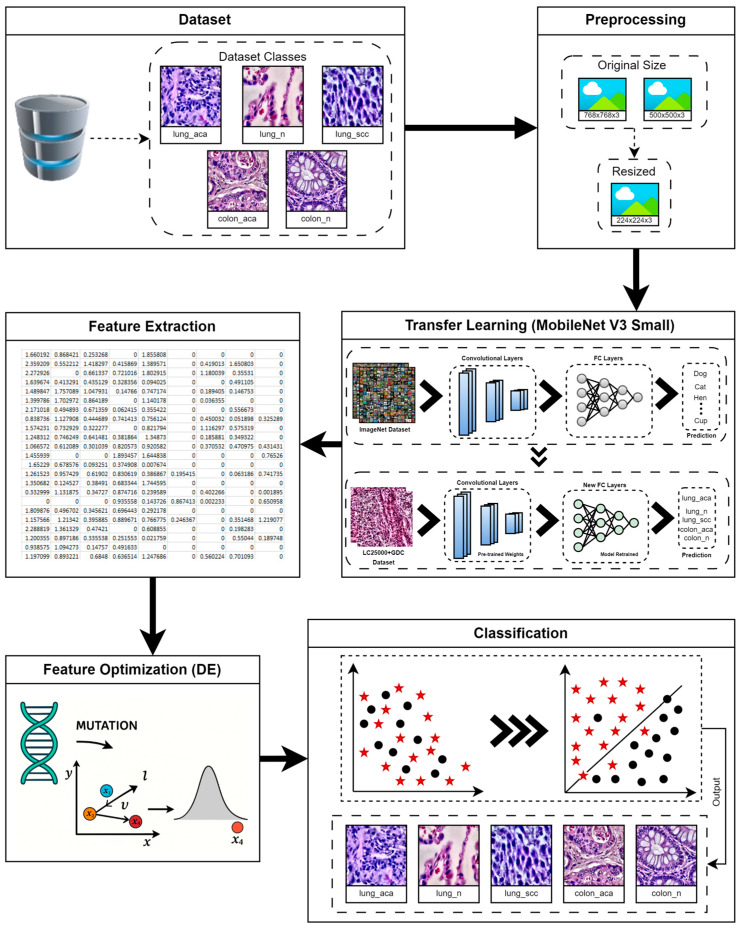
Proposed hybrid methodology for lung and colon classification using enhanced LC25000 dataset, where resized images are processed through MobileNetV3-Small for feature extraction and optimized using DE, and both original and optimized features are classified using NN-, SVM-, and tree-based models for comparative analysis.

This section presents the methodology proposed for this study, along with its detailed flow diagram, shown in Figure 1. In this study, the preprocessed LC25000 dataset, comprising five classes, was used. A transfer learning-based approach was proposed, with features extracted from the transfer-learned model. A differential evolution-based optimization technique is used to reduce and select the optimal features from the feature vector. Different classification models have been used to evaluate the proposed methodology, and results are gathered at every step.

### 2.1. Dataset

**Figure 2:**
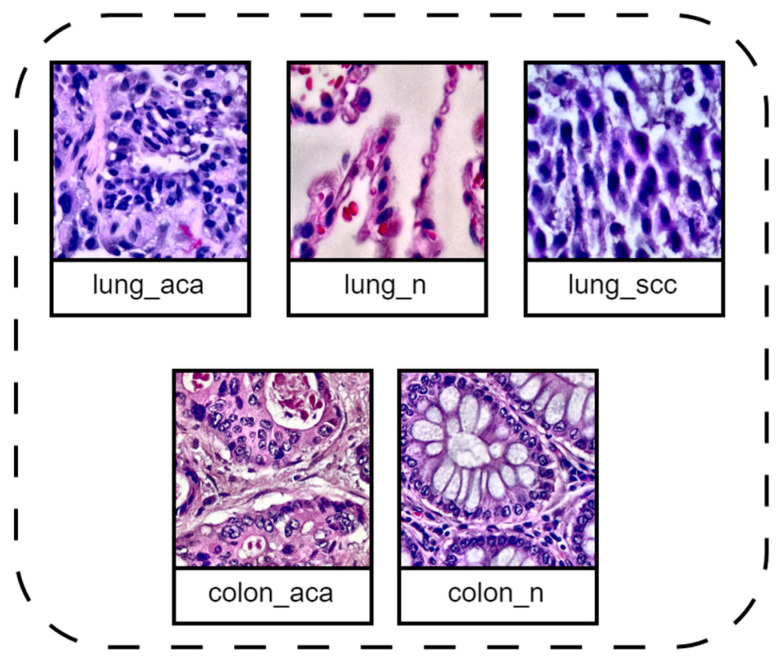
Representative histopathological sample from the dataset showing five classes, i.e., lung_aca, lung-n, lung_scc, colon_aca, and colon_n.

The dataset used consists of 25,000 histopathological images across 5 classes (benign colonic tissue, colon adenocarcinoma, lung adenocarcinoma, benign lung tissue, and lung squamous cell carcinoma), with 5000 images per class, gathered from [15]. Sample images of all five classes are shown in Figure 2. The original ‘LC25000’ dataset was created by augmenting 500 colon images (250 colon adenocarcinomas and 250 benign colon tissue) and 750 lung images (250 benign lung tissue, 250 lung squamous cell carcinomas, and 250 lung adenocarcinomas), which are the original samples of validated sources and HIPAA compliants [33]. These images are augmented to 5000 per class, resulting in each class having many patch redundancies. To improve robustness and clinical generalizability, 1000 images across three cancer classes (lung squamous cell carcinoma, lung adenocarcinoma, and colon adenocarcinoma) were replaced with cases from the NCI GDC data portal, selected based on a primary adenocarcinoma diagnosis by Ochoa-Ornelas, R. et al. [15]. Images were extracted using Aperio Image Scope [34] at 20×–40× magnification from diagnostic regions. These images were then preprocessed using CLAHE to improve the contrast of histopathological images. CLAHE enhances local contrast by applying histogram equalization within small image regions, making it well-suited for medical imaging as it highlights fine tissue details while avoiding noise over-amplification [35]. The dataset used in this research is listed in Table 1.

### 2.2. Transfer Learning

In deep learning, the dependence on a large volume of data was a significant challenge. To uncover complex and hidden patterns, a substantially larger volume of data is required to train deep learning models than for traditional machine learning models [36]. To overcome this issue, a transfer learning approach is used, in which a small dataset is used to pretrain the model by leveraging a knowledge base from the base domain to enhance the performance on the target objective.

TL is formally defined within a domain $H=\left[V,P\left(V\right)\right]$, where V and $P\left(V\right)$ denote the feature space and probability distribution, respectively. A task $T=\left[L,p\left(.\right)\right]$ consists of a label space L and a predictive function $p\left(.\right)$ learned from labeled pairs $\left[v_{i},l_{i}\right]$. The source domain $H_{s}=\left[\left(v_{s1},l_{s1}\right),\ldots ,\left(v_{sn},l_{sn}\right)\right]$ provides feature label pairs with prediction function $p_{s}\left(.\right)$, whereas the target domain $H_{T}=\left[\left(v_{T1},l_{T1}\right),\ldots ,\left(v_{Tn},l_{Tn}\right)\right]$ contains data along with its prediction function. Transfer learning aims to improve $p_{T}\left(.\right)$ for the target domain by leveraging knowledge from the source domain $H_{s}$.

#### MobileNet V3-Small

Transfer learning is performed on MobileNetV3-Small to extract features. Transfer learning with MobileNetV3-Small provides a lightweight yet robust backbone [37], offering fast inference, reduced computation cost, and strong accuracy on limited-data medical imaging tasks. To match the canonical dimension size of the MobileNetV3-Small, we resized the dataset images to a fixed size of 224 × 224 × 3 [38]. The model is initialized with ImageNet-pretrained weights. Given the image $x\in R^{3x224x224}$, the convolutional backbone $f_{\theta }$ produces feature maps using Equation (1):

(1) $$h=f_{\theta },\;h\in R^{576xH^{\prime }xW^{\prime }}$$

These are reduced to a fixed-length vector through global average pooling (GAP) using Equation (2):

(2) $$z=GAP\left(h\right)=\frac{1}{H^{\prime }W^{\prime }}\sum_{i=1}^{H^{\prime }}\sum_{j=1}^{W^{\prime }}h_{:,i,j},z\in R^{576}$$

The pooled feature vector is then projected into a higher-dimensional latent space via Equation (3):

(3) $$z_{1}=\sigma \left(BN\left(W_{1z}+b_{1}\right)\right),z_{1}\in R^{1536}$$

where $W_{1}\in R^{1536x576},\sigma \left(.\right)=ReLU\left(.\right),$ and dropout (p=0.3) is applied. This representation is subsequently compressed to Equation (4):

(4) $$z_{2}=\sigma \left(BN\left(W_{2z1}+b_{2}\right)\right),z_{2}\in R^{1024}$$

with $W_{2}\in R^{1024x1536}$ and dropout (p=0.3). The final classification layer maps this feature space to class probabilities, which is formulated as shown in Equation (5) below:

(5) $$\hat{y}=Softmax\left(W_{3z3}+b_{3}\right),W_{3}\in R^{cx1024}$$

where C is the number of output classes.

**Figure 3:**
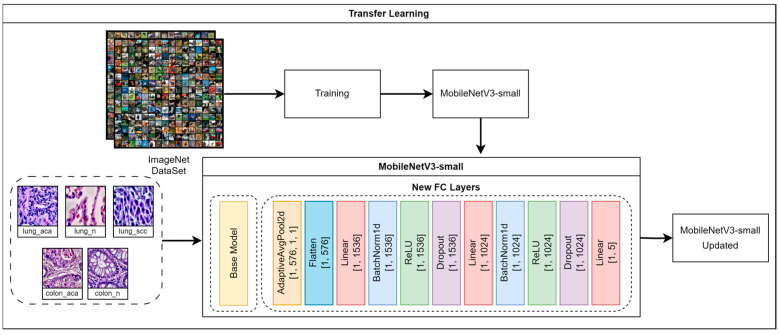
Overview of the transfer learning process using MobileNetV3-Small with added fully connected layers. The architecture is used to extract discriminative deep features from histopathological images.

During training, all backbone parameters θ were frozen, and only the newly introduced classification layers were optimized. The model was fine-tuned for 10 epochs on our dataset to adapt the classifier head. After training, the 1024-dimensional representation z2 (the second-to-last layer of the custom head) was extracted as the final feature vector for downstream analysis. Input images were preprocessed using the standard MobileNetV3-Small transform pipeline, including resizing to 224 × 224 and normalization with ImageNet statistics. Transfer learning process using MobileNetV3-Small has been shown in the Figure 3.

### 2.3. Differential Evolution

**Figure 4:**
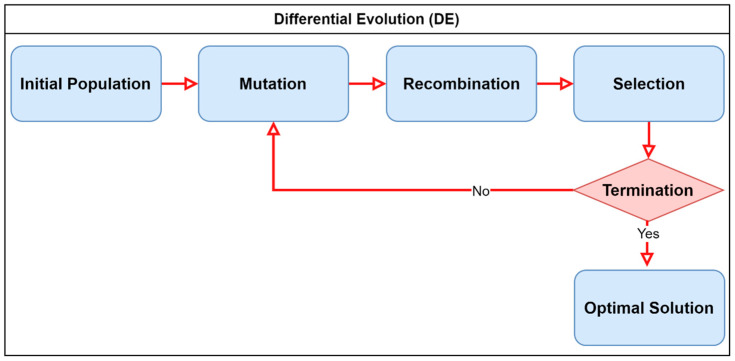
Flow diagram of the differential evolution algorithm illustrating population initialization, mutation, recombination, and selection. The process iteratively converges to an optimal feature subset.

Differential evolution (DE) is a population-based evolutionary optimizer inspired by the principles of Darwinian natural selection and biological evolution through mutation and recombination. To reduce and select the feature vector size, DE efficiently searches the solution space by iteratively evolving a population of candidate feature subsets, thus discarding redundant and irrelevant dimensions while retaining the discriminative features [39]. Compared to other classical optimization methods, such as gradient-based search or greedy feature ranking, DE does not rely on gradients, is less prone to local optima, and provides a balance between exploration (searching diverse subsets) and exploitation (refining promising candidates). Figure 4 shows a general flow diagram of the differential evolution optimizer working.

In this proposed methodology, we used a binary variant of DE for the feature selection. The algorithm maintains a population of binary candidate solutions, where each vector is represented as in Equation (6), which corresponds to a feature subset:

(6) $$x\;\varepsilon \;{\left\{0,1\right\}}^{D}$$

Each solution is evaluated by fitness using a custom cost function given in Equation (7):

(7) $$F\left(x\right)=\alpha .error\left(x\right)+\left(1-\alpha \right).\left(\frac{\left|x\right|}{D}\right)$$

where $\left|x\right|$ denotes the number of selected features and α balances prediction error and feature sparsity. The classification error term is bounded within [0, 1], while the sparsity term is expressed relative to the total number of features, ensuring a balanced contribution of both terms without requiring explicit normalization. The steps in Algorithm 1 are involved in this study.
**Algorithm 1:** Differential evolution**Input:** Feature matrix of size N × d, where d is the number of extracted deep features. **Output**: Optimal feature subset represented as a binary mask, yielding reduced feature matrix $N\;\times \;d_{opt}$.
(1)Step 1: Parameter Initialization
Population Size (*Pop*) = 10Iterations (*G_max_*) = 200Mutation factor (*F*) = 0.5Crossover rate (*Cr*) = 0.7Regularization weight $\left(\alpha \right)=0.005$Representation: Binary mask of dimension *d*.(2)Step 2: Population Initialization
Generate an initial population of size Pop X d using random binary values:$x_{i}^{j}=randint(0,2)$Each vector corresponds to a subset of selected features.(3)Step 3: Fitness Evaluation
For each individual, compute fitness using: $$Fitness=ClassificationError\left(X_{mask},y\right)+\alpha \times \frac{Number\;of\;selected\;features}{d}$$Where $X_{mask}$ is the reduced dataset after applying the binary mask.(4)Step 4: Mutation
For each $x_{i}$, choose three distinct vectors $x_{r1},x_{r2},x_{r3}.$Generate mutant vector:$$v_{i}^{j}=x_{r1}^{j}+F\times \left(x_{r2}^{j}-x_{r3}^{j}\right),\;v_{i}^{j}\in [0,1]$$
(5)Step 5: Crossover
Perform binomial crossover to form trial vector $u_{i}$:$$u_{i}^{j}=\left\{\begin{array}{c} v_{i}^{j}\;if\;rand\left(0,1\right)\leq Cr\;or\;j=j_{rand}\\ x_{i}^{j}\;otherwise \end{array}\right.$$Ensure at least one crossover point.Binarize trial vector: $$u_{i}^{j}=\left\{\begin{array}{c} 1\;if\;u_{i}^{j}>0.5\\ 0\;otherwise \end{array}\right.$$
(6)Step 6: Selection
Evaluate fitness of $u_{i}$.If $Fitness\left(u_{i}\right)<Fitness\left(x_{i}\right)$, replace: $$x_{i}=u_{i}$$Update global best solution if improved.
(7)Step 7: Termination
Repeat Steps 3–6 for $G_{max}=200$ iterations.Return best binary mask (optimal feature subset).**Result:** Optimal feature vector of reduced dimension $N\;\times \;d_{opt}$, where $d_{opt}<d$.

In this study, the parametric settings performed are shown in Table 2, along with a comparison with standard DE.

Instead of being adjusted for continuous optimization, the DE parameters were adjusted for feature selection in deep learning pipelines. To reduce computational load while preserving diversity, a small population (*Pop* = 10) and a moderate number of iterations (200) were used. By penalizing larger feature subsets, the regularization weight (*α* = 0.005) encourages sparsity and guides the optimizer to select only the most informative features.

#### Computational Cost Analysis

The computational cost analysis for DE was evaluated based on the execution time. The optimization process was performed on a 1024-dimensional feature vector, which required 42.8 s for 200 iterations under the selected parameter setting shown in Table 2. The reported computational time is hardware-dependent and is intended to provide a relative measure of efficiency within the proposed framework rather than a direct benchmark against other optimization techniques. The DE optimizer significantly reduced the feature dimension from 1024 to 60 features with a substantial reduction of 94.14% in feature space. This reduction in feature space improves the efficiency of the subsequent classification models by lowering the computational cost while preserving the discriminative information, which has been discussed in detail in Section 4 for this study.

### 2.4. Classification

For classification, two different feature vectors of dimensions N×1024 and N×60 are used, which are obtained from MobileNetV3-Small and differential evolution (DE) optimizers, respectively. These feature vectors are then split into 65% training, 15% validation, and 20% testing, which is shown in Table 3 using a stratified split with a fixed random seed (42), ensuring class balancing and reproducibility. Multiple classifiers from different families, such as NN, SVM, and DT, are used. Moreover, the results were also gathered using a commonly used split ratio of 80:10:10 for training, validation, and testing, after an optimal feature vector was obtained from a differential evolution optimizer, for comparison with the divided ratio 65:15:20.

We employed a diverse set of classifiers, including neural networks, support vector machines (SVMs), and ensemble-based tree learners. Neural networks are configured in two variants: a medium NN with hidden layers of 64 and 32 neurons, and a wide NN with larger hidden layers of 256 and 128 neurons, both using rectified linear unit (ReLU) activation and Softmax for multiclass classification [40]. These neural networks were designed with two hidden layers, as the input features are already high-level representations extracted from MobileNetV3-Small. This shallow architecture is therefore sufficient for effective classification while reducing the risk of overfitting and maintaining computational efficiency. SVMs are implemented with linear, quadratic, and cubic kernels to capture both simple and higher-order nonlinear decision boundaries. Moreover, ensemble approaches are utilized, including bagged trees with 50 base estimators, Gradient Boosting with 100 weak learners of depth three, and Adaboost employing decision stumps (depth = 1) with 100 iterations. This diverse selection ensured a balanced comparison across shallow classifiers, deep models, and ensemble learning paradigms, aligning with best practices in machine learning research.

## 3. Results

In the study, we used the LC25000 with the histopathological dataset from the GDC data portal for our experimentation. The original LC25000 dataset contains many near-duplicate patches. Using cases selected based on a primary adenocarcinoma diagnosis from the NCI GDC data portal, 1000 images across three cancer classes (lung squamous cell carcinoma, colon adenocarcinoma, and lung adenocarcinoma) were selected to increase robustness and clinical generalizability. This dataset is publicly available from Ochoa-Ornelas, R. et al. [15]. These images were then preprocessed using CLAHE to enhance the contrast of histopathological images.

In our preprocessing phase, the images in the dataset had two different dimensions: 768 × 768 × 3 and 500 × 500 × 3. Fixed dimension resizing of 224 × 224 × 3 pixels was performed to match the MobileNetV3-Small canonical input dimension size, which was used to transfer learn the model for feature extraction.

We computed results at every step of our proposed methodology: (a) with the use of a pretrained MobileNetV3-Small model, we extracted the feature vector of shape N × 1024 and then passed those to different classifiers to compute accuracies. (b) Then, we used the differential evolution optimizer, which gave us the optimal feature vector of shape N × 60 and passed it to classifiers to report a change in accuracy.

Different classifiers were used to compare the accuracies. The classifiers used in our study are adaboost trees, wide neural networks, linear SVM, bagged trees, boosted trees, quadratic SVM, cubic SVM, and medium neural networks. Multiple evaluation metrics are used to report results, including precision, sensitivity, accuracy, prediction time (sec), and area under the curve. Moreover, confidence intervals were computed using standard statistical estimation based on test set variability:

(8) $$Accuracy=\frac{True\;Positive+True\;Negative}{True\;Positive+True\;Negative+False\;Positive+False\;Negative}$$

(9) $$Precision=\frac{True\;Positive}{True\;Positive+False\;Positive}$$

(10) $$Sensitivity=\frac{True\;Positive}{True\;Positive+False\;Negative}$$

(11) $$F1-Score=\frac{2*Precision*Sensitivity}{Precision+Sensitivity}$$

The hyperparameters of the classifiers are selected based on standard configurations, with minor adjustments to ensure stable performance. Consistent settings were maintained across the experiments to allow fair comparison of the proposed methodology. The hypermeters used in our study are as follows in Table 4.

We used an Intel Core i5 12th-generation computer with Windows 11 Pro as the operating system and 32 GB of RAM to get the results. Python (v3.14.4) and Jupyter Notebook (v7.5.5) were utilized as the simulation and result-gathering environments.

**Figure 5:**
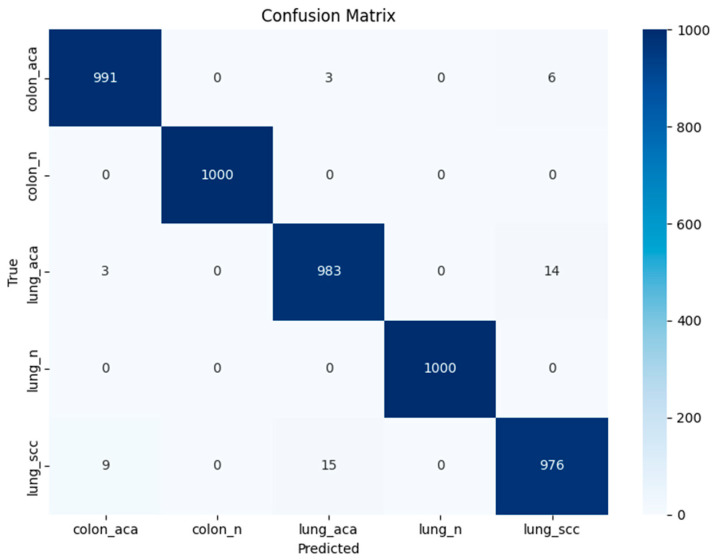
Confusion matrix for medium NN using deep features from MobileNetV3-Small shows that lung_scc and lung_acc exhibit minor misclassification, primarily confused with each other due to the same histopathological features, while all other classes are predicted with high accuracy.

Numerical results obtained from the original MobileNetV3-Small deep features are presented in Table 5. With an F1-score, recall, and precision of 0.9900, the medium neural network exhibits the highest accuracy of 0.9900. The misclassification rate of this classifier is 0.0100, and the AUC is 0.9998. The confidence interval for the medium neural network is 0.99 ± 0.0024. The confusion matrix and class-wise performance for a medium neural network are shown in Figure 5 and Table 6, respectively. With an F1-score, recall, and precision of 0.9898, the quadratic SVM exhibits the second-highest accuracy of 0.9898. The misclassification rate of this classifier is 0.0102, and the AUC is 0.9997. The confidence interval for the quadratic SVM is 0.9898 ± 0.0026. A vast neural network achieves the third-highest accuracy of 0.9897, with F1-scores, recalls, and precisions of 0.9897, 0.9896, and 0.9898, respectively. The misclassification rate of this classifier is 0.0103, and the AUC is 0.9998. The confidence interval for the vast neural network is 0.9897 ± 0.0025.

**Figure 6:**
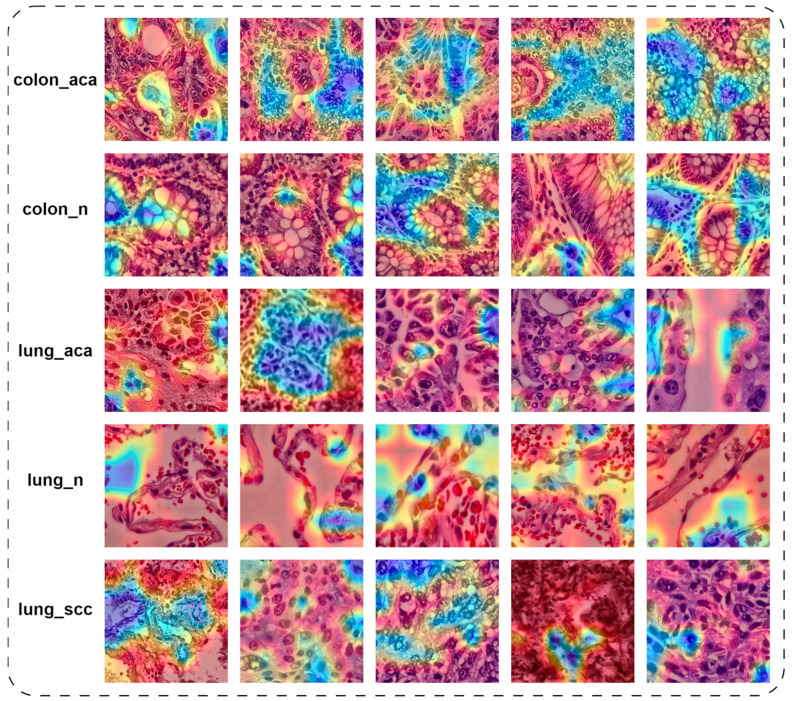
Grad-CAM visualizations showing regions contributing to model prediction across different classes. Highlighted areas correspond to diagnostically relevant histopathological structures.

Figure 6 illustrates the morphological features captured by the MobileNetV3-Small model on data images from each class. The visual analysis shows that for colon_aca, activations are focused on irregular glandular structure, whereas for colon_n, the focus is directed towards the well-organized glandular structure. Similarly, for lung_aca and lung_scc, activations are focused on dense tumor regions and abnormal cellular aggregation. However, for lung_n, focus is diverted towards the alveolar structures and thin tissue walls. This analysis shows that the model consistently concentrates on diagnostically significant regions.

**Figure 7:**
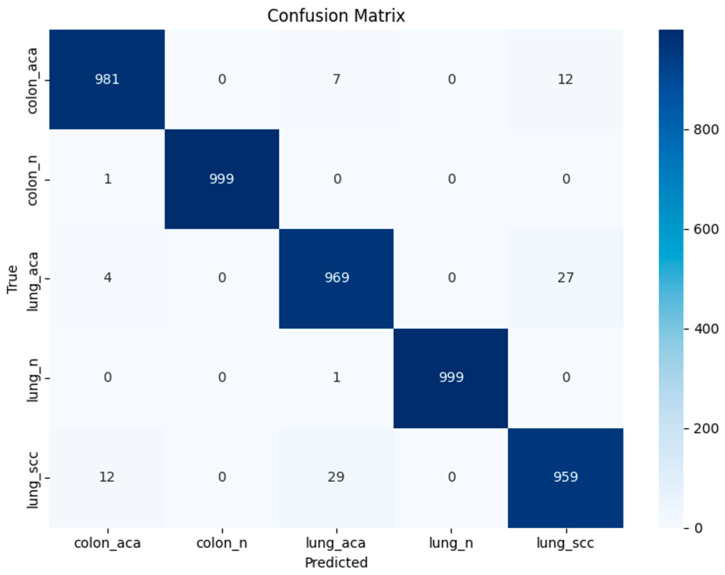
Confusion matrix for quadratic SVM using deep features after optimization shows that lung_scc and lung_acc exhibit minor misclassification primarily confused with each other due to same histopathological features, while all other classes are predicted with high accuracy.

The numerical results obtained after applying the differential evolution optimization are presented in Table 7. The quadratic SVM achieves the highest accuracy of 0.9814, with F1-score, recall, and precision of 0.9814. It shows the misclassification rate of 0.0186 and the AUC of 0.9993. The confidence interval for the quadratic SVM is 0.9814 ± 0.0034. The confusion matrix and class-wise performance for the quadratic SVM are shown in Figure 7 and Table 8, respectively. The cubic SVM ranks second, with F1-score, precision, recall, and accuracy of 0.9808. The misclassification rate of this classifier is 0.0192, and the AUC is 0.9992. The confidence interval for the cubic SVM is 0.9808 ± 0.0036. The medium neural network and linear SVM achieve the third-highest accuracy of 0.9804, with the neural network achieving F1-score, recall, and precision of 0.9804, 0.9804, and 0.9805, respectively. Moreover, the linear SVM achieves an F1-score, recall, and precision of 0.9804. The misclassification rate for both classifiers is 0.0196, with the AUC of 0.9994 and the confidence interval of 0.9804 ± 0.0038.

**Figure 8:**
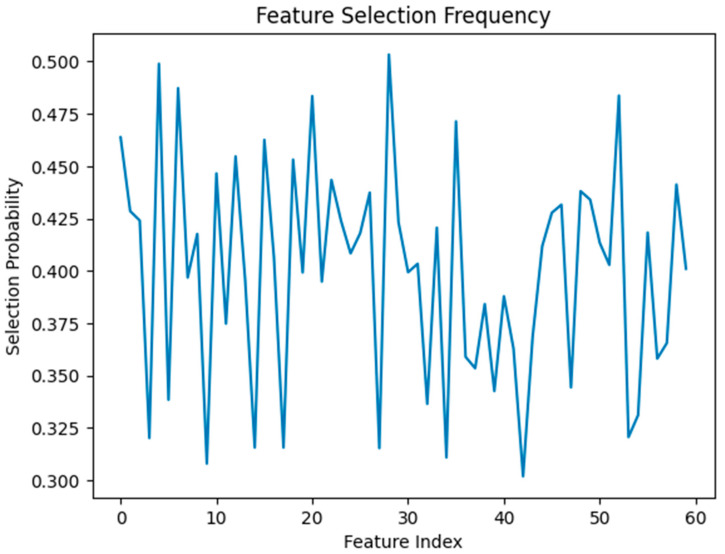
Feature selection frequency obtained using differential evolution, showing the probability of each feature being selected across iterations. The distribution indicates the relative importance of features in the optimized subset.

Figure 8 illustrates the feature selection frequency analysis, showing DE selects a diverse set of moderately informative features instead of only focusing on a small subset of dominant features. Rather than drastically reducing the features, the optimizer mainly focuses on multiple complementary characteristics. In histopathological analysis, where patterns are distributed across multiple feature dimensions, this behavior of DE is particularly significant.

We also reported results using an 80:10:10 training, validation, and test split, shown in Table 9, along with a comparison with the split used in this study. The comparison shows that using the proposed split results in a slight increase in accuracy for most classifiers, and in terms of prediction time, it provides a quite significant speed-up over the commonly used split. Moreover, the proposed split also used 20% of unseen data for testing, which better demonstrates the proposed methodology’s performance.

## 4. Discussions

Histopathological image classification for lung and colon cancer is challenging due to intra-class variability and inter-class similarity, compounded by staining and imaging variations. Deep learning methods, particularly CNN-based models, have shown strong capability in learning discriminative features. In this work, MobileNetV3-Small is utilized for efficient feature extraction, while differential evolution is applied to refine these features by reducing redundancy and enhancing class separability.

Table 10 presents a comparative analysis of classification accuracy and prediction time between the original MobileNetV3-Small deep features and those optimized using the differential evolution (DE) algorithm across various classifiers. Among all models, the medium neural network achieved the highest accuracy of 0.9900446 with the original features, followed closely by the vast neural network (0.989689) and the quadratic SVM (0.9898104). However, a slight decrease in accuracy was observed after DE optimization, with differences of −0.0096582 (medium neural network), −0.0123308 (vast neural network), and −0.0084362 (quadratic SVM).

**Figure 9:**
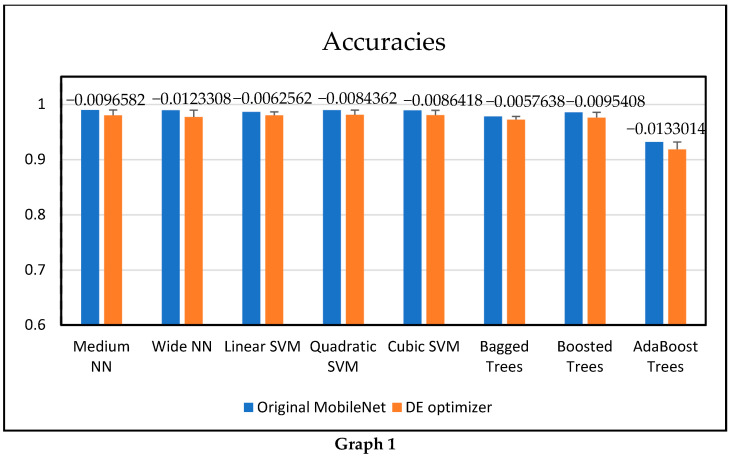

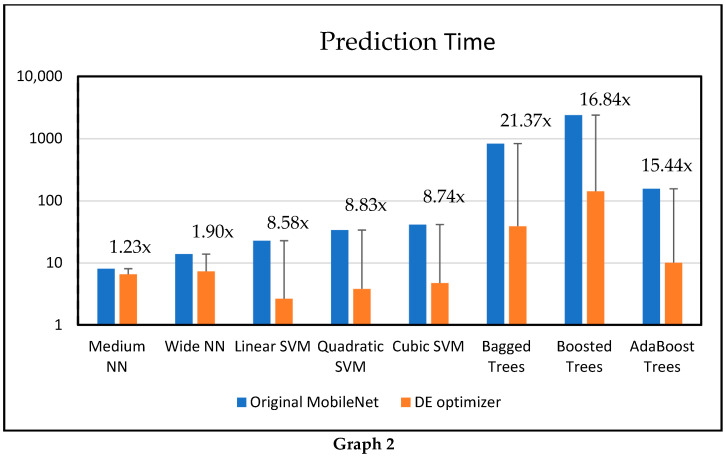
**Graph 1.** Comparison of classification accuracies for different classifiers using MobileNetV3-Small and DE optimized features. The results demonstrate comparable performance with slight variation after feature optimization. **Graph 2.** Comparison of prediction time across classifiers using MobileNetV3-Small and DE optimized features. The DE-based feature reduction significantly improves computational efficiency.

Despite this minor drop in accuracy, DE optimization significantly improved prediction speed. For instance, linear SVM and quadratic SVM achieved a speed-up factor of 8.58× and 8.83×, respectively. The most notable improvement is seen in the bagged tree, with a 21.37× speed-up, reducing prediction time from over 825 s to just 39 s. Similarly, boosted and adaboosted trees experienced substantial gains in efficiency, with 16.84× and 15.44× speed-ups, respectively. Neural networks benefited, though to a lesser extent, with the medium and wide neural networks achieving 1.23× and 1.90× faster prediction times, respectively. The visual representations of the accuracy difference and speed-up are shown in Figure 9 (Graph 1 and Graph 2), respectively.

The comparative results show that the optimized features consistently improve computational performance by retaining accuracy across multiple classifiers. This highlights the effectiveness of optimization with deep learning. This hybrid approach aligns with emerging trends in medical imaging, where the merging of optimization and deep feature learning techniques leads to generalizable and robust diagnostic methods.

A comparative analysis of the proposed methodology with other state-of-the-art techniques is shown in Table 11, where most of the work uses the LC25000 dataset, whereas this research uses the dataset from [15] by Ochoa-Ornelas, R. et al. The results obtained with the proposed technique are also compared with the dataset used by Ochoa-Ornelas, R., et al. In this enhanced dataset, histopathological images from the NCI GDC data portal are added to LC25000, whereas most previous work used only the LC25000 dataset. The LC25000 is a highly augmented dataset, where high accuracy is achieved across all metrics, suggesting that these models produce inflated performance metrics and fail to capture data patterns in real-world scenarios.

The proposed methodology achieved an accuracy of 98.14%, which represents a 3.34% increase over [15], which proposed an enhanced dataset. Compared with previous work using LC25000, the reported accuracy is slightly lower. The slightly lower accuracy achieved on the enhanced dataset shows that the results obtained offer a more realistic benchmark for clinical applications, rather than the accuracy obtained solely from LC25000, a highly augmented dataset. Moreover, most work only reports the accuracy achieved; we also included the prediction time to assess the model’s speed during training and testing. Only one paper using LC25000 reported the speed-up time achieved, i.e., 1.7×, whereas the proposed methodology shows a speed-up time of 21.37×, which is 19.67× higher. These results, compared with the state-of-the-art, demonstrate that the proposed method, using an enhanced dataset, outperforms in both speed and accuracy, whereas most articles do not focus on speed.

## 5. Conclusions and Future Work

This study presents an efficient automated framework for lung and colon cancer classification that addresses the limitations of traditional manual diagnostic approaches, including high cost, long processing time, and susceptibility to errors. An enhanced version of the LC25000 dataset is utilized, incorporating clinically relevant images from the National Cancer Institute GDC data portal while removing redundant patches to improve robustness and generalizability. The proposed approach integrates three key stages: (i) transfer learning using MobileNetV3-Small, (ii) optimal feature selection through differential evolution, and (iii) classification using multiple machine learning models. Comprehensive experiments conducted at each stage demonstrate that the proposed method achieves a strong trade-off between accuracy and computational efficiency. The best performance is obtained with a 98.14% accuracy using quadratic SVM and a 21.37× speed-up using bagged trees, representing a 3.34% improvement over the baseline study on the enhanced dataset. The use of a lightweight deep model combined with feature optimization makes the approach well-suited for practical clinical environments.

Future work will focus on further refining the enhanced LC25000 dataset by eliminating additional redundant patches and incorporating expert-validated clinical annotations. These enhancements are expected to improve dataset generalizability and support the development of more reliable and realistic benchmarks for real-world clinical deployment.

## Figures and Tables

**Table 1:** Class-wise distribution of the dataset showing the number of images obtained from the original LC25000 dataset and the GDC data portal, along with the total number of images for each histopathological class.

Class Name	Images from Original ‘LC25000’	Images from ‘GDC Data Portal’	Total Images
Colon_aca	4000	1000	5000
Colon_n	5000	-	5000
Lung_aca	4000	1000	5000
Lung_n	5000	-	5000
Lung_scc	4000	1000	5000

**Table 2:** Parametric configuration of standard DE with binary feature selection variant.

Parameters	Standard DE	Binary Feature Selection (Our Work)
Population size (Pop)	~10 × *problem dimension* (*d*) (e.g., 50–100)	10 (fixed, small for efficiency)
$\mathrm{Iteration}\;(G_{max})$	100–1000	200
Mutation factor (F)	0.4–1.0	0.5
Crossover rate (Cr)	0.7–0.9	0.7
Regularization weight (α)	Not used	0.005 (penalty for feature subset size)
Representation	Continuous real-valued vectors	Binary mask vectors {0, 1}
Fitness function	Benchmark functions (Sphere, Rosenbrock, etc.)	Classification error + sparsity penalty

**Table 3:** Training, validation, and testing splits.

Splits	Ratio	No. of Images
Training	65	17,000
Validation	15	3000
Testing	20	5000

**Table 4:** Classifiers key hyperparameters.

Classifier	Key Hyperparameters
Medium Neural Network (NN)/Wide Neural Network (NN)	Optimizer: Adam (lr = 0.001), Loss: Categorical Cross-Entropy, Activation: ReLU, Output: Softmax, Epochs: 10, Batch size: 32
Linear SVM	Kernel: linear, C = 1.0, γ = scale, Probability: True
Quadratic SVM	Kernel: poly, Degree: 2, C = 1.0, γ = scale, Coef0 = 0, Probability: True
Cubic SVM	Kernel: poly, Degree: 3, C = 1.0, γ = scale, Coef0 = 0, Probability: True
Bagged Trees	Base Estimator: DecisionTree (default: Gini, max_depth = None, min_samples_split = 2), n_estimators = 50, Bootstrap = True, Random_state = 42
Boosted Trees	n_estimators = 100, Subsample = 1.0, Criterion: Friedman MSE, Max_depth = 3, Random_state = 42, Learning_rate = 0.1
AdaBoost Trees	Base Estimator: DecisionTree(max_depth = 1, criterion = Gini), Random_state = 42, n_estimators = 100, Algorithm: SAMME.R, Learning_rate = 0.5

**Table 5:** Classification report along with CI, balanced accuracy, and Cohen’s Kappa scores using deep features from MobileNetV3-Small.

Model Name	Accuracy	Precision	Recall	F1-Score	Misclassification Rate	AUC	CI	Balanced Accuracy	Cohen’s Kappa
Medium NN	0.9900	0.9900	0.9900	0.9900	0.0100	0.9998	0.99 ± 0.0024	0.9874	0.98425
Quadratic SVM	0.9898	0.9898	0.9898	0.9898	0.0102	0.9997	0.9898 ± 0.0026	0.987	0.98375
Wide NN	0.9897	0.9898	0.9896	0.9897	0.0103	0.9998	0.9897 ± 0.0025	0.9884	0.9855
Cubic SVM	0.9894	0.9894	0.9894	0.9894	0.0106	0.9997	0.9894 ± 0.0028	0.9872	0.984
Linear SVM	0.9866	0.9866	0.9866	0.9866	0.0134	0.9997	0.9866 ± 0.0032	0.9846	0.98075
Boosted Trees	0.9858	0.9859	0.9858	0.9858	0.0142	0.9996	0.9858 ± 0.0032	0.9856	0.982
Bagged Trees	0.9784	0.9785	0.9784	0.9784	0.0216	0.9991	0.9784 ± 0.0038	0.982	0.9775
AdaBoost Trees	0.9320	0.9406	0.9320	0.9319	0.0680	0.9875	0.932 ± 0.0064	0.954	0.9425

**Table 6:** Class-wise performance for medium neural network using deep features from MobileNetV3-Small.

Classes	Precision	Sensitivity	F1-Score	Specificity
colon_aca	0.9910	0.9880	0.9895	0.9977
colon_n	1.0000	1.0000	1.0000	1.0000
lung_aca	0.9830	0.9820	0.9825	0.9957
lung_n	1.0000	1.0000	1.0000	1.0000
lung_scc	0.9760	0.9799	0.9780	0.9940

**Table 7:** Classification report along with CI, balanced accuracy, and Cohen’s Kappa using deep features after optimization.

Model Name	Accuracy	Precision	Recall	F1-Score	Misclassification Rate	AUC	CI	Balanced Accuracy	Cohen’s Kappa
Quadratic SVM	0.9814	0.9814	0.9814	0.9814	0.0186	0.9993	0.9814 ± 0.0034	0.9792	0.974
Cubic SVM	0.9808	0.9808	0.9808	0.9808	0.0192	0.9992	0.9808 ± 0.0036	0.976	0.97
Medium NN	0.9804	0.9805	0.9804	0.9804	0.0196	0.9994	0.9804 ± 0.0038	0.9778	0.97225
Linear SVM	0.9804	0.9804	0.9804	0.9804	0.0196	0.9994	0.9804 ± 0.0038	0.9812	0.9765
Wide NN	0.9774	0.9775	0.9774	0.9774	0.0226	0.9994	0.9774 ± 0.004	0.978	0.9725
Boosted Trees	0.9762	0.9762	0.9762	0.9762	0.0238	0.9993	0.9762 ± 0.0042	0.9764	0.9705
Bagged Trees	0.9726	0.9726	0.9726	0.9726	0.0274	0.9987	0.9726 ± 0.0042	0.9724	0.9655
AdaBoost Trees	0.9187	0.9220	0.9190	0.9199	0.0813	0.9863	0.9187 ± 0.0067	0.9376	0.922

**Table 8:** Class-wise performance for quadratic SVM using deep features after optimization.

Classes	Precision	Sensitivity	F1-Score	Specificity
colon_aca	0.9810	0.9830	0.9820	0.9953
colon_n	0.9990	1.0000	0.9995	0.9998
lung_aca	0.9690	0.9632	0.9661	0.9922
lung_n	0.9990	1.0000	0.9995	0.9998
lung_scc	0.9590	0.9609	0.9600	0.9898

**Table 9:** Proposed vs. commonly used split comparison over accuracy and prediction time.

Model Name	Commonly Used Split (80:10:10)		Proposed Split (65:15:20)		Diff in Accuracy	Speed-Up
	Accuracy	Prediction Time (s)	Accuracy	Prediction Time (s)		
Medium NN	0.9704172	9.9957813	0.9814	6.5985877	0.0109828	1.5148×
Wide NN	0.9736716	8.7247546	0.9808	7.3337453	0.0071284	1.1897×
Linear SVM	0.9724736	3.1790703	0.9804	2.6669071	0.0079264	1.192×
Quadratic SVM	0.9732468	3.9661065	0.9804	3.8435664	0.0071532	1.0319×
Cubic SVM	0.9712064	4.982683	0.9774	4.761102	0.0061936	1.0465×
Bagged Trees	0.9655664	29.8928554	0.9762	39.08775	0.0106336	0.7648×
Boosted Trees	0.9694948	126.85285	0.9726	142.697	0.0031052	0.889×
AdaBoost Trees	0.9237516	10.1582867	0.9187	10.150	−0.0050516	1.0008×

**Table 10:** Comparison of accuracy and prediction time for lung and colon cancer.

Classifiers	Accuracy			Prediction Time (s)		
	Original MobileNet	DE Optimizer	Accuracy Difference	Original MobileNet	DE Optimizer	Speed-Up
Medium NN	0.9900446	0.9803864	−0.0096582	8.1155656	6.5985877	1.23×
Wide NN	0.989689	0.9773582	−0.0123308	13.9372185	7.3337453	1.90×
Linear SVM	0.9866142	0.980358	−0.0062562	22.8873376	2.6669071	8.58×
Quadratic SVM	0.9898104	0.9813742	−0.0084362	33.9344222	3.8435664	8.83×
Cubic SVM	0.9894164	0.9807746	−0.0086418	41.6290729	4.761102	8.74×
Bagged Trees	0.9783836	0.9726198	−0.0057638	835.302611	39.08775	21.37×
Boosted Trees	0.9857754	0.9762346	−0.0095408	2403.476135	142.6972612	16.84×
AdaBoost Trees	0.932042	0.9187406	−0.0133014	156.7575045	10.1500119	15.44×

**Table 11:** A comparative analysis of the methodologies proposed on the ‘Enhanced LC25000’ dataset.

References	Dataset Used	Maximum Achieved Accuracy (%)	Precision	Recall	F1-Score	Maximum Achieved Speed-Up
Masud, M. et al. [8], 2021	LC25000	96.33	96.39	96.37	96.38	N/A
Mehmood, S. et al. [16], 2022	LC25000	98.4	N/A	N/A	N/A	N/A
Ijaz, M. et al. [18], 2023	LC25000	98.73	98.73	98.73	98.73	37.66% (1.7×)
Anjum, S. et al. [20], 2023	LC25000	97	N/A	N/A	N/A	N/A
Obayya, M. et al. [41], 2023	LC25000	99.33	98.31	98.31	98.31	N/A
Tummala, S. et al. [42], 2023	LC25000	99.97	99.97	99.97	99.97	N/A
Kumar, A. et al. [17], 2024	LC25000	98.92	99.64	99.84	99.65	N/A
Hasan, M. A. et al. [43], 2024	LC25000	99.20	99.16	99.36	99.16	N/A
Alotaibi, M. et al. [44], 2024	LC25000	99.6	99	99	99	N/A
Ochoa-Ornelas, R. et al. [15], 2024	LC25000 + National Cancer Institute GDC Data Portal	94.8	97.81	94.8	94.8	N/A
Proposed	LC25000 + National Cancer Institute GDC Data Portal	98.14	98.14	98.14	98.14	21.37×

## Data Availability

The data presented in this study are openly available on GitHub at https://github.com/AlbertoGudinoOchoa/Enhanced-LC25000-CLAHE-Dataset-Cancer-Classification (accessed on 2 December 2025) and Google Drive at https://drive.google.com/drive/folders/1aQNez61naAiuveaQlSzJ2VBsMI5_KUYm (accessed on 2 December 2025), by Ochoa-Ornelas, R. et al. [15].
